# Diethyl 3,4-bis­(2,5-dimethoxy­benz­yl)thieno[2,3-*b*]thio­phene-2,5-di­car­boxylate

**DOI:** 10.1107/S1600536809042214

**Published:** 2009-10-17

**Authors:** M. Umadevi, R. Sureshbabu, A. K. Mohanakrishnan, G. Chakkaravarthi, V. Manivannan

**Affiliations:** a Department of Chemistry, Pallavan College of Engineering, Kanchipuram 631 502, Tamilnadu, India; b Department of Organic Chemistry, University of Madras, Guindy Campus, Chennai 600 025, India; c Department of Physics, CPCL Polytechnic College, Chennai 600 068, India; d Department of Research and Development, PRIST University, Vallam, Thanjavur 613 403, Tamil Nadu, India

## Abstract

In the title compound, C_30_H_32_O_8_S_2_, the dihedral angle between the two benzene rings is 18.8 (1)°. The mol­ecular structure is stabilized by weak intra­molecular C—H⋯O hydrogen bonds. In the crystal structure, the mol­ecules are linked *via* weak inter­molecular C—H⋯O hydrogen bonds and π–π inter­actions between two benzene rings [centroid–centroid distance = 3.672 (1) Å].

## Related literature

For the biological activity of thio­phene derivatives, see: Tapia *et al.* (2003 ▶); Dallemagne *et al.* (2003 ▶). For related structures, see: Dufresne & Skene (2008 ▶); Gunasekaran *et al.* (2009 ▶). For graph-set notation, see: Bernstein *et al.* (1995 ▶)
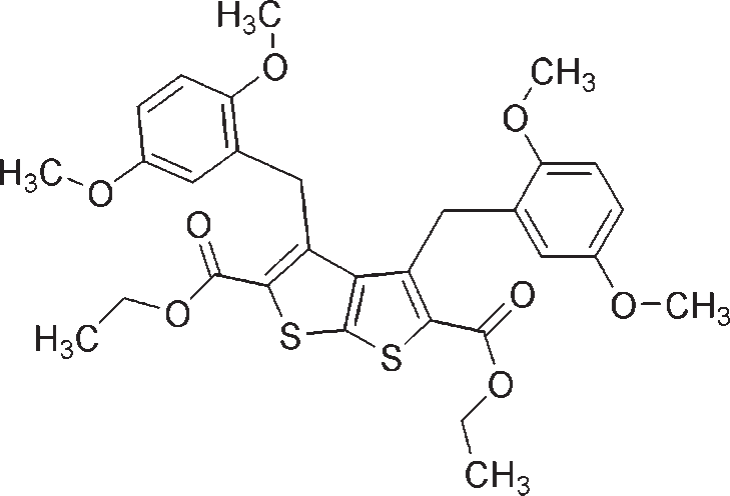


## Experimental

### 

#### Crystal data

C_30_H_32_O_8_S_2_
*M* *_r_* = 584.68
Triclinic, 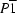

*a* = 9.9439 (3) Å
*b* = 10.8163 (3) Å
*c* = 14.7536 (5) Å
α = 82.610 (2)°
β = 89.490 (2)°
γ = 64.983 (1)°
*V* = 1424.20 (8) Å^3^
*Z* = 2
Mo *K*α radiation
μ = 0.24 mm^−1^
*T* = 295 K
0.24 × 0.20 × 0.18 mm

#### Data collection

Bruker Kappa APEX2 CCD diffractometer
Absorption correction: multi-scan (*SADABS*; Sheldrick, 1996 ▶) *T*_min_ = 0.945, *T*_max_ = 0.959
40341 measured reflections
11207 independent reflections
7512 reflections with *I* > 2σ(*I*)
*R*_int_ = 0.028

#### Refinement

*R*[*F* ^2^ > 2σ(*F* ^2^)] = 0.045
*wR*(*F* ^2^) = 0.130
*S* = 1.02
11207 reflections
367 parameters
H-atom parameters constrained
Δρ_max_ = 0.38 e Å^−3^
Δρ_min_ = −0.21 e Å^−3^

### 

Data collection: *APEX2* (Bruker, 2004 ▶); cell refinement: *SAINT* (Bruker, 2004 ▶); data reduction: *SAINT*; program(s) used to solve structure: *SHELXS97* (Sheldrick, 2008 ▶); program(s) used to refine structure: *SHELXL97* (Sheldrick, 2008 ▶); molecular graphics: *PLATON* (Spek, 2009 ▶); software used to prepare material for publication: *SHELXL97*.

## Supplementary Material

Crystal structure: contains datablocks global, I. DOI: 10.1107/S1600536809042214/bt5095sup1.cif

Structure factors: contains datablocks I. DOI: 10.1107/S1600536809042214/bt5095Isup2.hkl

Additional supplementary materials:  crystallographic information; 3D view; checkCIF report

## Figures and Tables

**Table 1:** Hydrogen-bond geometry (Å, °)

*D*—H⋯*A*	*D*—H	H⋯*A*	*D*⋯*A*	*D*—H⋯*A*
C13—H13*B*⋯O2	0.97	2.43	2.9923 (17)	117
C22—H22*B*⋯O4	0.97	2.38	3.0169 (16)	123
C17—H17⋯O2^i^	0.93	2.43	3.3267 (16)	162
C25—H25⋯O4^ii^	0.93	2.51	3.2280 (16)	134

Symmetry codes: (i) 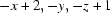; (ii) 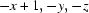.

**Fig. 1.**
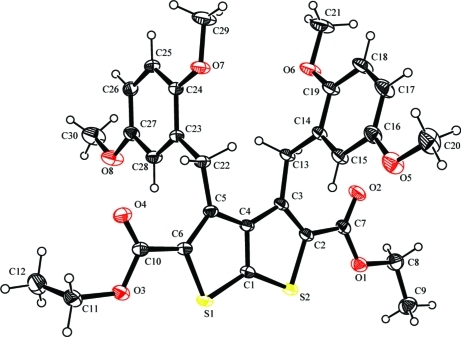
The molecular structure of the title compound, with atom labels and 30% probability displacement ellipsoids for non-H atoms.

**Fig. 2.**
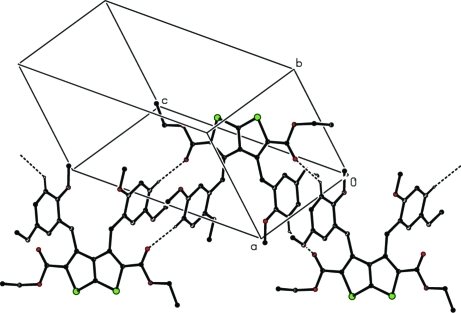
C—H···O hydrogen bonds of the title compound. Hydrogen bonds are shown as dashed lines.

## supplementary crystallographic information

### Comment

In continuation of our studies on thiophene derivatives which exhibit biological activities such as anti-protozoal (Tapia *et al.*, 2003), antitumor (Dallemagne *et al.*, 2003), we report the crystal structure of the title compound. The geometric parameters of the title compound (Fig. 1) agree with the reported similar structures (Dufresne & Skene, 2008; Gunasekaran *et al.*, 2009). The dihedral angle between the phenyl rings C14—C19 and C23—C28 is 18.8 (1)°.

The molecular structure is stabilized by intramolecular C—H···O hydrogen bonds. In the crystal structure, the molecules are linked *via* intermolecular C—H···O hydrogen bonds (see, Fig. 2), and are further consolidated by   π···π [*Cg*4···*Cg*4 (1 - *x*, -*y*, -*z*) = 3.672 (1) Å; *Cg*4 is the centroid of C23—C28 ring] interactions. The intermolecular C17—H17···O2 hydrogen bond generates a twenty-membered ring, with a graph-set motif of *R*_2_^2^(20) (Bernstein *et al.*, 1995).

### Experimental

To a solution of Diethyl 3,4-bis-(acetoxymethyl) thieno[2,3,-b]thiophene-2,5-dicarboxylate (0.7 g, 1.63 mmol) in dry 1,2-dichloroethane (10 ml), Ferric Chloride (0.05 g, 0.32 mmol) and 1,4-dimethoxybenzene (0.54 g, 3.92 mmol) were added under nitrogen atmosphere. The reaction mixture was refluxed for 20 hr, then it was poured over crushed ice (40 g) containing 1 ml of Conc.HCl. Ferric chloride was carefully filtered off and the filtrate was evaporated under reduced pressure giving crude product and it was crystallized from methanol affording pure product.

### Refinement

H atoms were positioned geometrically and refined using a riding model with C—H = 0.93Å and *U*_iso_(H) = 1.2Ueq(C) for aromatic C—H, with C—H = 0.97Å and *U*_iso_(H) = 1.2Ueq(C) for methylene and C—H = 0.96Å and *U*_iso_(H) = 1.5Ueq(C) for methyl.

### Crystal data

**Table:**

C_30_H_32_O_8_S_2_	*Z* = 2
*M**_r_* = 584.68	*F*(000) = 616
Triclinic, *P*1	*D*_x_ = 1.363 Mg m^−^^3^
Hall symbol: -P 1	Mo *K*α radiation, λ = 0.71073 Å
*a* = 9.9439 (3) Å	Cell parameters from 7622 reflections
*b* = 10.8163 (3) Å	θ = 2.3–32.6°
*c* = 14.7536 (5) Å	µ = 0.24 mm^−^^1^
α = 82.610 (2)°	*T* = 295 K
β = 89.490 (2)°	Block, colourless
γ = 64.983 (1)°	0.24 × 0.20 × 0.18 mm
*V* = 1424.20 (8) Å^3^	

### Data collection

**Table:**

Bruker Kappa APEX2 CCD diffractometer	11207 independent reflections
Radiation source: fine-focus sealed tube	7512 reflections with *I* > 2σ(*I*)
graphite	*R*_int_ = 0.028
ω and φ scans	θ_max_ = 33.6°, θ_min_ = 1.4°
Absorption correction: multi-scan (*SADABS*; Sheldrick, 1996)	*h* = −15→13
*T*_min_ = 0.945, *T*_max_ = 0.959	*k* = −16→16
40341 measured reflections	*l* = −22→22

### Refinement

**Table:**

Refinement on *F*^2^	Primary atom site location: structure-invariant direct methods
Least-squares matrix: full	Secondary atom site location: difference Fourier map
*R*[*F*^2^ > 2σ(*F*^2^)] = 0.045	Hydrogen site location: inferred from neighbouring sites
*wR*(*F*^2^) = 0.130	H-atom parameters constrained
*S* = 1.01	*w* = 1/[σ^2^(*F*_o_^2^) + (0.0618*P*)^2^ + 0.2259*P*] where *P* = (*F*_o_^2^ + 2*F*_c_^2^)/3
11207 reflections	(Δ/σ)_max_ < 0.001
367 parameters	Δρ_max_ = 0.38 e Å^−^^3^
0 restraints	Δρ_min_ = −0.21 e Å^−^^3^

### Fractional atomic coordinates and isotropic or equivalent isotropic displacement parameters (Å^2^)

**Table:**

	*x*	*y*	*z*	*U*_iso_*/*U*_eq_	
C1	0.33884 (12)	0.45771 (12)	0.29435 (9)	0.0319 (2)	
C2	0.45021 (11)	0.29918 (12)	0.43341 (9)	0.0311 (2)	
C3	0.50197 (11)	0.23469 (11)	0.35827 (8)	0.0279 (2)	
C4	0.43974 (11)	0.32916 (11)	0.27586 (8)	0.0279 (2)	
C5	0.45557 (11)	0.32473 (11)	0.17974 (8)	0.0281 (2)	
C6	0.36170 (13)	0.44795 (12)	0.13047 (9)	0.0325 (2)	
C7	0.48843 (12)	0.24180 (13)	0.53018 (9)	0.0352 (3)	
C8	0.4489 (2)	0.30018 (16)	0.68064 (10)	0.0521 (4)	
H8A	0.5547	0.2632	0.6956	0.062*	
H8B	0.4148	0.2303	0.7021	0.062*	
C9	0.3674 (2)	0.42647 (18)	0.72430 (12)	0.0566 (4)	
H9A	0.3969	0.4971	0.6991	0.085*	
H9B	0.3904	0.4057	0.7891	0.085*	
H9C	0.2625	0.4580	0.7128	0.085*	
C10	0.33664 (14)	0.48317 (12)	0.03113 (9)	0.0363 (3)	
C11	0.17568 (19)	0.64930 (15)	−0.08706 (10)	0.0504 (4)	
H11A	0.2622	0.6182	−0.1238	0.060*	
H11B	0.1266	0.7491	−0.0991	0.060*	
C12	0.07259 (19)	0.5918 (2)	−0.11375 (13)	0.0612 (4)	
H12A	0.1226	0.4930	−0.1047	0.092*	
H12B	0.0408	0.6239	−0.1770	0.092*	
H12C	−0.0124	0.6213	−0.0768	0.092*	
C13	0.60622 (11)	0.08518 (11)	0.36206 (9)	0.0307 (2)	
H13A	0.5851	0.0515	0.3086	0.037*	
H13B	0.5867	0.0342	0.4156	0.037*	
C14	0.76908 (11)	0.05525 (12)	0.36583 (8)	0.0309 (2)	
C15	0.81823 (12)	0.15692 (13)	0.35845 (10)	0.0371 (3)	
H15	0.7493	0.2487	0.3492	0.045*	
C16	0.96928 (13)	0.12566 (15)	0.36447 (10)	0.0424 (3)	
C17	1.07178 (14)	−0.00949 (16)	0.37866 (12)	0.0522 (4)	
H17	1.1727	−0.0313	0.3835	0.063*	
C18	1.02365 (14)	−0.11288 (15)	0.38571 (12)	0.0520 (4)	
H18	1.0930	−0.2045	0.3950	0.062*	
C19	0.87411 (13)	−0.08212 (13)	0.37913 (10)	0.0389 (3)	
C20	1.15560 (18)	0.2084 (2)	0.36269 (15)	0.0663 (5)	
H20A	1.1985	0.1574	0.4214	0.099*	
H20B	1.1659	0.2933	0.3551	0.099*	
H20C	1.2059	0.1550	0.3154	0.099*	
C21	0.92037 (18)	−0.31882 (15)	0.40392 (16)	0.0665 (5)	
H21A	0.9815	−0.3431	0.3525	0.100*	
H21B	0.8675	−0.3755	0.4136	0.100*	
H21C	0.9818	−0.3329	0.4576	0.100*	
C22	0.56168 (12)	0.20106 (11)	0.13954 (9)	0.0306 (2)	
H22A	0.6533	0.1583	0.1773	0.037*	
H22B	0.5850	0.2314	0.0791	0.037*	
C23	0.50319 (12)	0.09487 (11)	0.13174 (8)	0.0284 (2)	
C24	0.60272 (12)	−0.03646 (11)	0.11506 (8)	0.0303 (2)	
C25	0.55198 (14)	−0.13381 (12)	0.10341 (9)	0.0347 (3)	
H25	0.6187	−0.2204	0.0913	0.042*	
C26	0.40264 (14)	−0.10439 (13)	0.10943 (9)	0.0365 (3)	
H26	0.3693	−0.1709	0.1016	0.044*	
C27	0.30409 (13)	0.02372 (13)	0.12702 (10)	0.0368 (3)	
C28	0.35525 (13)	0.12273 (12)	0.13736 (9)	0.0341 (3)	
H28	0.2879	0.2098	0.1483	0.041*	
C29	0.85392 (16)	−0.19303 (15)	0.10302 (15)	0.0629 (5)	
H29A	0.8512	−0.2543	0.1555	0.094*	
H29B	0.9515	−0.1954	0.1000	0.094*	
H29C	0.8306	−0.2214	0.0485	0.094*	
C30	0.09936 (18)	−0.03504 (18)	0.13132 (16)	0.0695 (6)	
H30A	0.1141	−0.0652	0.0721	0.104*	
H30B	−0.0048	0.0039	0.1422	0.104*	
H30C	0.1515	−0.1122	0.1774	0.104*	
O1	0.41917 (11)	0.34007 (10)	0.58310 (7)	0.0441 (2)	
O2	0.57034 (11)	0.12509 (10)	0.55927 (7)	0.0472 (2)	
O3	0.22256 (12)	0.60698 (10)	0.00899 (7)	0.0478 (2)	
O4	0.40665 (12)	0.41188 (10)	−0.02441 (7)	0.0465 (2)	
O5	1.00283 (11)	0.23685 (12)	0.35686 (9)	0.0599 (3)	
O6	0.81740 (10)	−0.17839 (10)	0.38624 (9)	0.0527 (3)	
O7	0.74872 (9)	−0.05753 (9)	0.11046 (7)	0.0422 (2)	
O8	0.15417 (11)	0.06544 (10)	0.13456 (9)	0.0567 (3)	
S1	0.25637 (3)	0.57151 (3)	0.19869 (2)	0.03757 (9)	
S2	0.32384 (3)	0.47150 (3)	0.40793 (2)	0.03599 (8)	

### Atomic displacement parameters (Å^2^)

**Table:**

	*U*^11^	*U*^22^	*U*^33^	*U*^12^	*U*^13^	*U*^23^
C1	0.0286 (5)	0.0222 (5)	0.0380 (6)	−0.0045 (4)	−0.0017 (4)	−0.0035 (5)
C2	0.0230 (4)	0.0262 (5)	0.0381 (6)	−0.0055 (4)	−0.0013 (4)	−0.0014 (5)
C3	0.0186 (4)	0.0217 (5)	0.0395 (6)	−0.0059 (4)	−0.0011 (4)	−0.0009 (4)
C4	0.0219 (4)	0.0202 (5)	0.0391 (6)	−0.0061 (4)	−0.0003 (4)	−0.0049 (4)
C5	0.0260 (5)	0.0193 (5)	0.0389 (6)	−0.0090 (4)	0.0005 (4)	−0.0058 (4)
C6	0.0346 (5)	0.0215 (5)	0.0380 (6)	−0.0082 (4)	−0.0005 (5)	−0.0058 (5)
C7	0.0258 (5)	0.0351 (6)	0.0410 (7)	−0.0103 (4)	−0.0003 (4)	−0.0022 (5)
C8	0.0692 (10)	0.0439 (8)	0.0348 (7)	−0.0176 (7)	−0.0034 (7)	0.0002 (6)
C9	0.0710 (10)	0.0516 (9)	0.0457 (9)	−0.0236 (8)	0.0054 (7)	−0.0103 (7)
C10	0.0423 (6)	0.0236 (5)	0.0406 (7)	−0.0119 (5)	−0.0031 (5)	−0.0032 (5)
C11	0.0619 (9)	0.0350 (7)	0.0397 (8)	−0.0092 (6)	−0.0058 (6)	0.0049 (6)
C12	0.0542 (9)	0.0628 (11)	0.0544 (10)	−0.0139 (8)	−0.0071 (7)	−0.0045 (8)
C13	0.0217 (4)	0.0215 (5)	0.0438 (7)	−0.0053 (4)	−0.0014 (4)	−0.0009 (5)
C14	0.0207 (4)	0.0281 (5)	0.0375 (6)	−0.0051 (4)	−0.0006 (4)	−0.0011 (5)
C15	0.0253 (5)	0.0309 (6)	0.0500 (8)	−0.0089 (4)	−0.0035 (5)	0.0016 (5)
C16	0.0282 (5)	0.0422 (7)	0.0562 (9)	−0.0161 (5)	−0.0016 (5)	0.0002 (6)
C17	0.0221 (5)	0.0527 (9)	0.0744 (11)	−0.0104 (5)	−0.0014 (6)	−0.0026 (8)
C18	0.0233 (5)	0.0375 (7)	0.0813 (11)	−0.0014 (5)	−0.0018 (6)	−0.0013 (7)
C19	0.0250 (5)	0.0287 (6)	0.0546 (8)	−0.0041 (4)	−0.0006 (5)	−0.0021 (6)
C20	0.0417 (8)	0.0801 (13)	0.0891 (14)	−0.0394 (9)	0.0021 (8)	−0.0048 (10)
C21	0.0420 (8)	0.0258 (7)	0.1165 (17)	−0.0014 (6)	0.0003 (9)	−0.0035 (8)
C22	0.0275 (5)	0.0214 (5)	0.0413 (7)	−0.0080 (4)	0.0039 (4)	−0.0080 (5)
C23	0.0305 (5)	0.0195 (5)	0.0325 (6)	−0.0079 (4)	−0.0004 (4)	−0.0040 (4)
C24	0.0312 (5)	0.0224 (5)	0.0333 (6)	−0.0074 (4)	−0.0010 (4)	−0.0040 (4)
C25	0.0398 (6)	0.0213 (5)	0.0392 (7)	−0.0086 (4)	−0.0008 (5)	−0.0066 (5)
C26	0.0436 (6)	0.0259 (6)	0.0423 (7)	−0.0169 (5)	−0.0042 (5)	−0.0041 (5)
C27	0.0325 (5)	0.0290 (6)	0.0489 (8)	−0.0136 (5)	−0.0018 (5)	−0.0029 (5)
C28	0.0318 (5)	0.0234 (5)	0.0454 (7)	−0.0095 (4)	0.0026 (5)	−0.0070 (5)
C29	0.0359 (7)	0.0305 (7)	0.1111 (16)	−0.0009 (6)	0.0019 (8)	−0.0191 (8)
C30	0.0446 (8)	0.0443 (9)	0.1278 (18)	−0.0277 (7)	−0.0021 (9)	−0.0076 (10)
O1	0.0496 (5)	0.0361 (5)	0.0352 (5)	−0.0082 (4)	−0.0011 (4)	−0.0010 (4)
O2	0.0403 (5)	0.0374 (5)	0.0461 (6)	−0.0019 (4)	−0.0060 (4)	0.0038 (4)
O3	0.0566 (6)	0.0305 (5)	0.0384 (5)	−0.0021 (4)	−0.0070 (4)	−0.0016 (4)
O4	0.0572 (6)	0.0320 (5)	0.0414 (5)	−0.0092 (4)	0.0003 (4)	−0.0094 (4)
O5	0.0373 (5)	0.0541 (7)	0.0934 (9)	−0.0274 (5)	−0.0039 (5)	0.0010 (6)
O6	0.0291 (4)	0.0249 (4)	0.0941 (9)	−0.0031 (3)	−0.0010 (5)	−0.0033 (5)
O7	0.0295 (4)	0.0246 (4)	0.0677 (7)	−0.0052 (3)	0.0021 (4)	−0.0119 (4)
O8	0.0339 (5)	0.0339 (5)	0.1055 (10)	−0.0176 (4)	0.0040 (5)	−0.0095 (6)
S1	0.03985 (16)	0.02006 (14)	0.03953 (18)	0.00000 (11)	−0.00335 (12)	−0.00368 (12)
S2	0.03251 (14)	0.02638 (15)	0.03817 (17)	−0.00160 (11)	0.00029 (11)	−0.00594 (12)

### Geometric parameters (Å, °)

**Table:**

C1—C4	1.3882 (15)	C16—O5	1.3691 (17)
C1—S2	1.7010 (13)	C16—C17	1.373 (2)
C1—S1	1.7028 (12)	C17—C18	1.383 (2)
C2—C3	1.3658 (17)	C17—H17	0.9300
C2—C7	1.4664 (18)	C18—C19	1.3810 (17)
C2—S2	1.7440 (11)	C18—H18	0.9300
C3—C4	1.4367 (16)	C19—O6	1.3713 (16)
C3—C13	1.5009 (15)	C20—O5	1.4175 (17)
C4—C5	1.4302 (17)	C20—H20A	0.9600
C5—C6	1.3736 (16)	C20—H20B	0.9600
C5—C22	1.5011 (15)	C20—H20C	0.9600
C6—C10	1.4622 (18)	C21—O6	1.4182 (16)
C6—S1	1.7434 (12)	C21—H21A	0.9600
C7—O2	1.1993 (15)	C21—H21B	0.9600
C7—O1	1.3411 (16)	C21—H21C	0.9600
C8—O1	1.4438 (17)	C22—C23	1.5070 (15)
C8—C9	1.489 (2)	C22—H22A	0.9700
C8—H8A	0.9700	C22—H22B	0.9700
C8—H8B	0.9700	C23—C28	1.3753 (16)
C9—H9A	0.9600	C23—C24	1.3980 (15)
C9—H9B	0.9600	C24—O7	1.3735 (14)
C9—H9C	0.9600	C24—C25	1.3749 (17)
C10—O4	1.2038 (16)	C25—C26	1.3856 (18)
C10—O3	1.3402 (15)	C25—H25	0.9300
C11—O3	1.4489 (17)	C26—C27	1.3738 (18)
C11—C12	1.484 (3)	C26—H26	0.9300
C11—H11A	0.9700	C27—O8	1.3709 (15)
C11—H11B	0.9700	C27—C28	1.3904 (17)
C12—H12A	0.9600	C28—H28	0.9300
C12—H12B	0.9600	C29—O7	1.4132 (16)
C12—H12C	0.9600	C29—H29A	0.9600
C13—C14	1.5113 (14)	C29—H29B	0.9600
C13—H13A	0.9700	C29—H29C	0.9600
C13—H13B	0.9700	C30—O8	1.4126 (18)
C14—C15	1.3723 (17)	C30—H30A	0.9600
C14—C19	1.3976 (16)	C30—H30B	0.9600
C15—C16	1.3940 (16)	C30—H30C	0.9600
C15—H15	0.9300		
			
C4—C1—S2	113.79 (9)	C16—C17—H17	120.3
C4—C1—S1	113.60 (10)	C18—C17—H17	120.3
S2—C1—S1	132.60 (7)	C19—C18—C17	120.97 (13)
C3—C2—C7	128.19 (11)	C19—C18—H18	119.5
C3—C2—S2	114.17 (9)	C17—C18—H18	119.5
C7—C2—S2	117.65 (9)	O6—C19—C18	124.55 (12)
C2—C3—C4	110.44 (10)	O6—C19—C14	115.56 (10)
C2—C3—C13	124.37 (11)	C18—C19—C14	119.89 (12)
C4—C3—C13	125.17 (11)	O5—C20—H20A	109.5
C1—C4—C5	111.91 (10)	O5—C20—H20B	109.5
C1—C4—C3	111.79 (11)	H20A—C20—H20B	109.5
C5—C4—C3	136.30 (10)	O5—C20—H20C	109.5
C6—C5—C4	110.87 (10)	H20A—C20—H20C	109.5
C6—C5—C22	125.33 (11)	H20B—C20—H20C	109.5
C4—C5—C22	123.80 (10)	O6—C21—H21A	109.5
C5—C6—C10	128.19 (11)	O6—C21—H21B	109.5
C5—C6—S1	113.52 (10)	H21A—C21—H21B	109.5
C10—C6—S1	118.23 (9)	O6—C21—H21C	109.5
O2—C7—O1	123.98 (13)	H21A—C21—H21C	109.5
O2—C7—C2	126.05 (13)	H21B—C21—H21C	109.5
O1—C7—C2	109.97 (10)	C5—C22—C23	113.91 (9)
O1—C8—C9	106.89 (12)	C5—C22—H22A	108.8
O1—C8—H8A	110.3	C23—C22—H22A	108.8
C9—C8—H8A	110.3	C5—C22—H22B	108.8
O1—C8—H8B	110.3	C23—C22—H22B	108.8
C9—C8—H8B	110.3	H22A—C22—H22B	107.7
H8A—C8—H8B	108.6	C28—C23—C24	118.43 (10)
C8—C9—H9A	109.5	C28—C23—C22	122.87 (10)
C8—C9—H9B	109.5	C24—C23—C22	118.67 (10)
H9A—C9—H9B	109.5	O7—C24—C25	124.38 (10)
C8—C9—H9C	109.5	O7—C24—C23	115.46 (10)
H9A—C9—H9C	109.5	C25—C24—C23	120.15 (10)
H9B—C9—H9C	109.5	C24—C25—C26	120.79 (11)
O4—C10—O3	123.61 (13)	C24—C25—H25	119.6
O4—C10—C6	125.62 (12)	C26—C25—H25	119.6
O3—C10—C6	110.77 (11)	C27—C26—C25	119.58 (11)
O3—C11—C12	111.27 (13)	C27—C26—H26	120.2
O3—C11—H11A	109.4	C25—C26—H26	120.2
C12—C11—H11A	109.4	O8—C27—C26	125.18 (11)
O3—C11—H11B	109.4	O8—C27—C28	115.27 (11)
C12—C11—H11B	109.4	C26—C27—C28	119.55 (11)
H11A—C11—H11B	108.0	C23—C28—C27	121.49 (11)
C11—C12—H12A	109.5	C23—C28—H28	119.3
C11—C12—H12B	109.5	C27—C28—H28	119.3
H12A—C12—H12B	109.5	O7—C29—H29A	109.5
C11—C12—H12C	109.5	O7—C29—H29B	109.5
H12A—C12—H12C	109.5	H29A—C29—H29B	109.5
H12B—C12—H12C	109.5	O7—C29—H29C	109.5
C3—C13—C14	114.58 (9)	H29A—C29—H29C	109.5
C3—C13—H13A	108.6	H29B—C29—H29C	109.5
C14—C13—H13A	108.6	O8—C30—H30A	109.5
C3—C13—H13B	108.6	O8—C30—H30B	109.5
C14—C13—H13B	108.6	H30A—C30—H30B	109.5
H13A—C13—H13B	107.6	O8—C30—H30C	109.5
C15—C14—C19	118.60 (10)	H30A—C30—H30C	109.5
C15—C14—C13	122.91 (10)	H30B—C30—H30C	109.5
C19—C14—C13	118.49 (10)	C7—O1—C8	116.59 (11)
C14—C15—C16	121.43 (12)	C10—O3—C11	116.74 (11)
C14—C15—H15	119.3	C16—O5—C20	116.70 (13)
C16—C15—H15	119.3	C19—O6—C21	117.14 (11)
O5—C16—C17	124.94 (12)	C24—O7—C29	116.74 (11)
O5—C16—C15	115.38 (12)	C27—O8—C30	117.02 (12)
C17—C16—C15	119.67 (13)	C1—S1—C6	90.05 (6)
C16—C17—C18	119.44 (12)	C1—S2—C2	89.71 (6)
			
C7—C2—C3—C4	−178.22 (11)	C13—C14—C19—O6	0.95 (18)
S2—C2—C3—C4	1.40 (12)	C15—C14—C19—C18	0.8 (2)
C7—C2—C3—C13	3.19 (18)	C13—C14—C19—C18	−178.01 (14)
S2—C2—C3—C13	−177.18 (8)	C6—C5—C22—C23	−97.49 (14)
S2—C1—C4—C5	−176.41 (8)	C4—C5—C22—C23	82.76 (13)
S1—C1—C4—C5	2.66 (13)	C5—C22—C23—C28	16.39 (17)
S2—C1—C4—C3	3.39 (13)	C5—C22—C23—C24	−165.78 (11)
S1—C1—C4—C3	−177.54 (8)	C28—C23—C24—O7	−179.86 (11)
C2—C3—C4—C1	−3.00 (13)	C22—C23—C24—O7	2.21 (16)
C13—C3—C4—C1	175.58 (10)	C28—C23—C24—C25	0.90 (18)
C2—C3—C4—C5	176.74 (12)	C22—C23—C24—C25	−177.03 (11)
C13—C3—C4—C5	−4.7 (2)	O7—C24—C25—C26	179.78 (12)
C1—C4—C5—C6	−2.46 (14)	C23—C24—C25—C26	−1.05 (19)
C3—C4—C5—C6	177.81 (12)	C24—C25—C26—C27	0.2 (2)
C1—C4—C5—C22	177.32 (10)	C25—C26—C27—O8	179.97 (13)
C3—C4—C5—C22	−2.4 (2)	C25—C26—C27—C28	0.8 (2)
C4—C5—C6—C10	−175.76 (12)	C24—C23—C28—C27	0.12 (19)
C22—C5—C6—C10	4.5 (2)	C22—C23—C28—C27	177.95 (12)
C4—C5—C6—S1	1.26 (13)	O8—C27—C28—C23	179.79 (12)
C22—C5—C6—S1	−178.52 (9)	C26—C27—C28—C23	−1.0 (2)
C3—C2—C7—O2	−2.2 (2)	O2—C7—O1—C8	0.9 (2)
S2—C2—C7—O2	178.21 (11)	C2—C7—O1—C8	−178.40 (11)
C3—C2—C7—O1	177.11 (11)	C9—C8—O1—C7	176.97 (12)
S2—C2—C7—O1	−2.51 (14)	O4—C10—O3—C11	5.4 (2)
C5—C6—C10—O4	−7.8 (2)	C6—C10—O3—C11	−174.08 (12)
S1—C6—C10—O4	175.33 (11)	C12—C11—O3—C10	83.66 (17)
C5—C6—C10—O3	171.65 (12)	C17—C16—O5—C20	1.1 (2)
S1—C6—C10—O3	−5.24 (15)	C15—C16—O5—C20	−179.98 (15)
C2—C3—C13—C14	−89.64 (14)	C18—C19—O6—C21	1.7 (2)
C4—C3—C13—C14	91.98 (13)	C14—C19—O6—C21	−177.22 (15)
C3—C13—C14—C15	−5.37 (18)	C25—C24—O7—C29	−7.0 (2)
C3—C13—C14—C19	173.40 (12)	C23—C24—O7—C29	173.75 (14)
C19—C14—C15—C16	−0.4 (2)	C26—C27—O8—C30	5.4 (2)
C13—C14—C15—C16	178.40 (13)	C28—C27—O8—C30	−175.40 (15)
C14—C15—C16—O5	−179.37 (13)	C4—C1—S1—C6	−1.63 (10)
C14—C15—C16—C17	−0.4 (2)	S2—C1—S1—C6	177.21 (10)
O5—C16—C17—C18	179.61 (16)	C5—C6—S1—C1	0.17 (10)
C15—C16—C17—C18	0.8 (3)	C10—C6—S1—C1	177.51 (10)
C16—C17—C18—C19	−0.3 (3)	C4—C1—S2—C2	−2.18 (9)
C17—C18—C19—O6	−179.35 (16)	S1—C1—S2—C2	178.98 (10)
C17—C18—C19—C14	−0.5 (3)	C3—C2—S2—C1	0.39 (9)
C15—C14—C19—O6	179.78 (13)	C7—C2—S2—C1	−179.94 (10)

### Hydrogen-bond geometry (Å, °)

**Table:**

*D*—H···*A*	*D*—H	H···*A*	*D*···*A*	*D*—H···*A*
C13—H13B···O2	0.97	2.43	2.9923 (17)	117
C22—H22B···O4	0.97	2.38	3.0169 (16)	123
C17—H17···O2^i^	0.93	2.43	3.3267 (16)	162
C25—H25···O4^ii^	0.93	2.51	3.2280 (16)	134

Symmetry codes: (i) −*x*+2, −*y*, −*z*+1; (ii) −*x*+1, −*y*, −*z*.
